# Iran health insurance system in transition: equity concerns and steps to achieve universal health coverage

**DOI:** 10.1186/s12939-020-01372-4

**Published:** 2021-01-14

**Authors:** Leila Doshmangir, Mohammad Bazyar, Arash Rashidian, Vladimir Sergeevich Gordeev

**Affiliations:** 1 Social Determinants of Health Research Center, Health Management & Safety Promotion Research Institute, Tabriz University of Medical Sciences, Tabriz, Iran; 2 Department of Health Policy& Management, Tabriz Health Services Management Research Center, Iranian Center of Excellence in Health Management, School of Management& Medical Informatics, Tabriz University of Medical Sciences, Tabriz, Iran; 3 Department of Public Health, Faculty of Health, Ilam University of Medical Sciences, Ilam, Iran; 4 Department of Science, Information and Dissemination, World Health Organization Regional Office for the Eastern Mediterranean, Cairo, Egypt; 5 Institute of Population Health Sciences, Queen Mary University of London, London, UK; 6 Department of Infectious Disease Epidemiology, London School of Hygiene & Tropical Medicine, London, UK

**Keywords:** Health insurance, Health policy and systems research, Health financing, Reform, Iran

## Abstract

**Background:**

Equity, efficiency, sustainability, acceptability to clients and providers, and quality are the cornerstones of universal health coverage (UHC). No country has a single way to achieve efficient UHC. In this study, we documented the Iranian health insurance reforms, focusing on how and why certain policies were introduced and implemented, and which challenges remain to keep a sustainable UHC.

**Methods:**

This retrospective policy analysis used three sources of data: a comprehensive and chronological scoping review of literature, interviews with Iran health insurance policy actors and stakeholders, and a review of published and unpublished official documents and local media. All data were analysed using thematic content analysis.

**Results:**

Health insurance reforms, especially health transformation plan (HTP) in 2014, helped to progress towards UHC and health equity by expanding population coverage, a benefits package, and enhancing financial protection. However, several challenges can jeopardize sustaining this progress. There is a lack of suitable mechanisms to collect contributions from those without a regular income. The compulsory health insurance coverage law is not implemented in full. A substantial gap between private and public medical tariffs leads to high out-of-pocket health expenditure. Moreover, controlling the total health care expenditures is not the main priority to make keeping UHC more sustainable.

**Conclusion:**

To achieve UHC in Iran, the Ministry of Health and Medical Education and health insurance schemes should devise and follow the policies to control health care expenditures. Working mechanisms should be implemented to extend free health insurance coverage for those in need. More studies are needed to evaluate the impact of health insurance reforms in terms of health equity, sustainability, coverage, and access.

## Introduction

The Sustainable Development Goals (SDGs) Summit during the opening week of the 74th session of the United Nations General Assembly gave a clear message that many countries are off-track to achieve many of the SDGs by 2030. Universal health coverage (UHC) is no exception [1, 2]. Some advocated for revisiting how healthcare sectors are being financed and suggested forgoing private health insurance as the least sustainable and equitable methods of healthcare financing. Others stressed that besides primary health care (PHC) and UHC, social health insurance (SHI) should be given priority in all and not just low- and middle-income countries (LMICs) [3, 4].

Many LMICs contemplate adopting SHI as it is one of the essential routes towards achieving UHC, besides general tax revenue as the primary source of financing health services. SHI can provide a stable source of revenues by combining risk pooling and mutual support with the visible flow of funds into the healthcare sector [5]. Since SHI, in principle, involves compulsory membership among all the population, in theory, its adoption should foster moving from existing partial health insurance coverage to UHC. Despite the positive experience of reaching UHC via social or universal health insurance implementation in several countries (i.e., South Korea, Thailand, Turkey) [6–8], the adaptation of their experience verbatim (including insurance scheme model and implementation strategy) would deem unsuccessful without accounting for particular country’s context. Contextual factors include, but are not limited to, existing population coverage and population needs, size of formal and informal healthcare sectors, cultural and political context, access or lack thereof to health care services, dependency ratios, demographics, population and geo-spatial characteristics, income-level differentials, administrative capability, political commitment and allocated or available financing [9]. Despite being known, these factors remain vastly understudied. While it is essential to localise and adapt previously successful experiences of others, it is equally important to account for reasons and factors why other countries with similar socio-economic and political context failed to implement SHI and achieve or sustain UHC. Only by learning from both would allow to take concrete steps in the development of SHI coverage that would lead to tangible results.

During the past nine decades, numerous reforms took place in the Iranian health insurance system intending to achieve UHC. However, despite several major attempts to extend health insurance coverage to the entire population, there are still several challenges to reach and keep a sustainable UHC. Evidence suggests that despite the introduction of the universal basic benefits package under the SHI fund in Iran, inequalities in health financing indicators and access to health care services continue to exist, particularly for low-income groups and rural residents [10, 11]. Some of the reforms that contributed towards achieving UHC were previously analysed (e.g., Family Physician program, PHC, hospital autonomy, and Health Transformation Plan implementation [3, 12–19]); however, more studies need to evaluate the impact of the health insurance reforms implemented in Iran.

Health policymaking is a complex process and suffers from limited uptake of research evidence, particularly in the Eastern Mediterranean Region. This study, aimed at generating insights about how health insurance policies were made over 90 years, identifies factors influencing policymaking and assesses to what extent these policies contributed to achieving UHC in Iran. Lessons learned from this study can be useful for informing future policymaking in health systems and provides insights for structuring the evidence-informed decision-making process, particularly in LMICs.

### Country context

Iran is an upper-middle-income country, located in the Middle East. It is the 17th largest country in the world with a population density of 51 people per km^2^. The median age in Iran is 30.1 years, of which more than 70% are urban dwellers [20]. Iran’s population is young, and almost one-third of the population is less than 15 years old, and only about 5% are over 60 years. According to the last survey (2015), the mean health literacy level in Iran was 10.2±3.8 (out of 20) [21].

The Iranian healthcare system has a well-defined three-tier structure, comprising primary, secondary, and tertiary facilities. In 2016, Iran spent 8.1% GDP on health (the equivalent of US$ 415.4 per capita), with a share of out-of-pocket (OOP) expenditure falling from 80.5% (in 1995) to 38.8% (in 2016) of current health expenditure [22]. The sources of funds to finance health care services are multiple and include (besides OOP payments) government funds, general taxation, health insurance, and individual donations. During the last decades, Iran has implemented remarkable initiatives to strengthen PHC to achieve UHC. PHC is provided nationwide free-of-charge to all Iranian citizens by public/private partnership and coordinated/regulated by the Ministry of Health and Medical Education (MoHME). The secondary health care is delivered via a network of district health clinics, which is an independent authority from the MoHME, provided by hospitals and coordinated/regulated by treatment deputy of medical universities under the supervision of MoHME. Tertiary health services are provided predominantly in big cities by private and public hospitals and coordinated/regulated by MoHME [3, 17].

Based on their functional nature, there are three main groups of organisations that provide health insurance coverage in Iran: SHI, institutional health insurance funds, and commercial organisations (Table 1). Table 2 shows the main features of different institutional and other health insurance schemes in Iran.

**Table 1:** Current health insurance funds in Iran

Type	Definition	Examples
**Social Health Insurance (SHI) schemes**	SHI provides basic health insurance coverage for their beneficiaries and includes three main insurance funds.	- **Iran Health Insurance Organization (IHIO)** IHIO includes five sub-funds 1. Governmental Employees Fund; 2. Iranian Fund for self-employed; 3. Rural Residents Fund (i.e., residents in rural areas and cities less than 20 000 population); 4. Universal Insurance Coverage Fund, which was established as part of a Health Transformation Plan in 2014 to cover uninsured persons; and 5. Other Sectors Fund (i.e., such as the poor, students, disables, families with injured persons during the war, and some professional associations). - **Social Security Organization (SSO)** SSO is a non-governmental organisation covering employees of the formal private sectors, self-employed and voluntary contributors. - **Armed Forces Medical Services Insurance Organization** This fund is responsible for covering special social cases and the army.
**Institutional Health Insurance Funds (IHIFs)**	These funds provide health insurance coverage to their employees individually as a fringe benefit.	17 IHIFs are run by the Petroleum Industry Health Organization, the National Broadcasting Organization, banks, and other organisations that provide the required insurance services to their employees.
**Commercial Health Insurance Organisations**	These organisations operate voluntarily and provide supplementary private insurance.	Examples of such funds include Alborz, Mellat, Pasargadae, Atieh Sazane Hafez.

**Table 2:** Main features of health insurance schemes in Iran (2018)

	IHIO				SSO	Armed Forces Health Insurance	
Features	Government employees	Rural residents	Universal Coverage	Other sectors			
Affiliation	Governmental	Governmental	Governmental	Governmental	Public Non-Governmental	Governmental	
Who is insured?	Government employees	Residents in rural areas and cities less than 20.000 population	Self-employed	Students, disables, families with injured persons during the war, some professional associations and similar	Employees of the formal private sectors, self-employed and voluntary contributors	Military personnel and their families	
Membership	Obligatory	Voluntary	Voluntary	Voluntary	Obligatory	Obligatory	
Population size (2018)	5.413.088	19.969.227	14.441.544	1.438.973	43.475.548 (2019)	Not available	
Contribution rate (2017)	7% of the wage (2%, 2% and 3% paid by the employee, employer, and government respectively)	7% of the minimum wage (paid by the government)	Fixed premium (400,000 Rial per month, 100% paid by the government)	7% of the minimum wage (100% paid by the government)	30% of which 9% is for health benefits package (2%, 6% and 1% paid by the worker, employer, and government respectively)	7% of the wage (2%, 2.5% and 2.5% paid by the employee, employer, and government respectively), An extra mandatory fixed amount is also deducted for supplementary coverage which is paid by the employees	
Per capita expenditures per year (2018)^26^	480,968 R*	277.565 R	371,580 R	573.173 R	431.719 (2019) R	Not available	
User charges	10 to 30% of in patient and out-patients health services respectively in the public hospitals based on public medical tariffs. Also, the gap between private and public medical tariffs in private centres	10 to 30%	10 to 30%	10 to 30%	10 to 30% of in patient and out-patients health services respectively based on public medical tariffs. Also, the gap between private and public tariffs in private centres. No copayment in SSO’s hospitals and health centres		

*R= Rial (Iran currency)

## Methods

### Study design

We conducted a retrospective policy analysis of the health insurance development in the Iranian health system. It is a historical analysis and interpretation of past policies. We used three sources of data: a comprehensive and chronological scoping review of literature, interviews with Iran health insurance policy actors and stakeholders; and a review of published and unpublished official documents and local media.

### Review of literature, documents, and local media

Three international (EMBASE, PubMed and Scopus) and two national (Magiran and Scientific Information Database which publish Iranian papers in Persian) bibliographical databases were searched for purposes of a scoping review. The following main search terms were used: health, insurance, policy interventions, and Iran. Also, references of all the final included papers were searched for articles not identified through electronic searches. Studies were included if they reported on the health insurance system in Iran, stakeholders, milestones, challenges, achievements and the policies effects on public health outcomes such as financial coverage, population coverage and health services coverage. There were no time restrictions for the search of papers or related documents. The publication language was restricted to English and Persian. The search was conducted between Aug 2019 and Oct 2020. Results from the bibliographic databases were merged, and duplicates were removed. We used narrative synthesis to present results. All related information was inductively extracted through the documentary review. Each article was reviewed by two reviewers (MB and LD), and information was extracted to fit the aims of the study.

Additionally, published and unpublished official reports by the national and international organisations (i.e., MoHME, basic health insurance organisations, social security organisation research centre, Iran’s Parliament Research Centre, World Health Organization, and the World Bank) were searched and reviewed. We searched the local official websites (i.e., health insurance organization, MoHME, Iran’ Parliament) and relevant data were extracted.

Extracted data were analyzed through content analysis. For document and media analysis, the data were examined and interpreted in order to elicit meaning, gain understanding, and develop empirical knowledge [23]. The analysis yielded data, including excerpts, quotations, or entire passages. Data were then organised into categories of health insurance trends and case examples.

### Qualitative interviews

Two key informant groups from MoHME and health insurance organization were identified to assist the research team in purposefully sampling of the interviewees. Twenty-six persons were interviewed from July to November 2017 (first round). Additional six persons were interviewed during September 2020 (second round). The second round was exploratory and aimed to clarify some ambiguities in the development of health insurance policies. Table 3 shows the characteristics of interviewees. All participants consented for participation. Interviews took place either face to face and via telephone, using an interview guide and a semi-structured form. The questions were determined in a flexible format which allowed emerging issues to be pursued. All interviews lasted about an hour, were recorded and transcribed verbatim. Transcriptions were analysed using qualitative content analysis methods of data reduction, data display and conclusion drawing/verification [24]. Data were “reduced” through searching patterns, major periods and milestones in health insurance development, reviewing subjects’ response to the questions in the interview guide and emerging questions in response to removing any ambiguity. We used the “Consolidated criteria for reporting qualitative research (COREQ): a 32-item checklist for interviews and focus groups” to certify that a high-quality report would be obtained.

**Table 3:** The characteristics of interviewees

Setting	Organizations, Institutions, political groups, and individuals	No.
Health Insurance System	The Ministry of Cooperatives, Labor and Social Welfare including the High Council of the Health Insurance	**3**
	Iran Health Insurance Organization	**9**
	Social Security Organization	**4**
	Armed Forces Health Insurance	**2**
	Imam Khomeini Relief Committee	**1**
Ministry of Health and Medical Education (MoHME)	Health Technology Assessment (HTA), Standard and Tariff Office	**4**
	Supreme Council for Health Policy Making	**3**
Supervisory/regulatory Organizations	Planning and Budget Organization	**2**
	Parliament Research Center	**1**
Medical Sciences Universities	Academician familiar with Iranian health system and health financing concepts	**3**
***Sum***		***32***

## Results

**Fig. 1:**
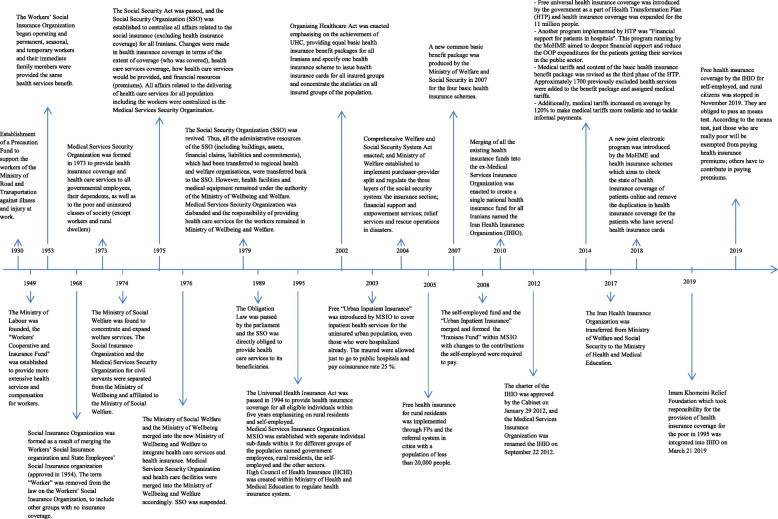
Key milestones in health insurance development in Iran

Our review included 117 articles, 22 reports and 112 other documents, including Parliament proceedings, gazettes, newsweekly issued by health insurance organisations, MoHME, Iran Parliament, and other organizations (e.g., Medical Council of the Islamic Republic of Iran). The synthesised evidence and findings regarding the evolution of the Iranian health insurance system fell into six major periods, based on the main steps undertaken to reach UHC: introduction of the first insurance scheme for workers (1930–1972); the birth of national health insurance organisations (1973–1983); organisational solidarity in health services stewardship (1984–1993); passing universal health insurance bill (1994–2003); extending health insurance coverage to all rural residents (2004–2013); and extending health insurance coverage to all residents (2014–2020) (Fig. 1). Majority of identified documents addressed three last periods.

### Chronological view of the key health insurance events in Iran, 1930–2019, is shown in Fig. 1

### Period 1– introduction of the first insurance scheme for workers (1930 to 1972)

In 1930, a first insurance scheme in Iran (a Precaution Fund) was introduced to protect workers of the Ministry of Road and Transportation against illnesses and occupational injuries at work [25–28]. In 1941, the Ministry of Wellbeing (Vezarat-e Behdari) was established. In 1943, the first mandatory law on workers’ insurance was passed, and they became insured by the Iran Insurance Company with a premium contribution set at 1.5–3% of their salary. This law extended insurance coverage to include disability and death at work, as well as introduced the practice of monitoring of working conditions. In 1946, the labour law expanded coverage further to include protection against unemployment, disability, and early retirements, as well as non-occupational diseases. In 1949, the Ministry of Labour and a new insurance fund (the Workers’ Cooperation and Insurance Fund or Sandoogh-e Taavon va Bime Kargaran) were established. It took over the responsibility of providing social insurance for the workers from the Iran Insurance Company with the premium rate being increased to 6%. The insurance included additional protection against non-occupational accidents, workers’ ageing and disability, provided additional financial aid for marriage, pregnancy, loss of the household’s head, and paid up to seven days per year of employment in the event of dismissal [26, 27]. Three years later, in 1952, the Fund was replaced by Workers Social Insurance Organization (WSIO) (Sazman-e Bime Ejtemaie Karkaran). After the “Workers’ Social Insurance Bill” was passed, all seasonal and temporary workers (including their immediate family members) were required to pay health insurance premiums. Health insurance premiums increased from 6 to 12% of the salary. Apart from providing social insurance coverage, WSIO was assigned to provide health services for the workers as well. In 1955, the insurance premium increased to 18%, and all buildings and health care facilities that had been established by governmental institutions for the regular use of workers were transferred to WSIO.

In 1968, after a merger of State Employee Social Insurance (SESI) and WSIO, the term “worker” was removed from a new title (becoming “Social Insurance Organization” or SIO) to reflect its newly extended coverage for all state employees and other workers without any insurance coverage. Additional attempts to expand workers insurance coverage were made by establishing a Rural Residents Social Insurance Fund (1969) to cover those working in rural areas. Although owing to administrative problems and a lack of effective operational policies, these organisations operated independently until 2001 [26, 29].

### Period 2 - the birth of national health insurance organisations (1973 to 1983)

In 1973, following the “Health Care for Civil Servants” law, the Medical Services Security Organization (MSSO) (Sazman Tamin-e Khadamate Darmani) was formed to fulfil two missions: to provide health insurance coverage for all government employees and to provide health care services to all employees of ministries, public institutions, state-owned enterprises and government companies, whether employed or retired, their family members, and other dependents, as well as to the poor and uninsured classes of society (except workers and rural dwellers) [26, 27]. One year later in 1974, the Ministry of Social Welfare (Vezarat-e Refahe Ejtemaie) was established, and it took over from the Ministry of Wellbeing (Vezarat-e Behdari) the administration of the provision of health insurance through SIO and MSSO. Statute of health insurance for armed forces in Iran was introduced in 1974.

In 1975, after the Social Security Act was passed, the Social Security Organization (SSO) was established and assigned to take over from Ministry of Social Welfare the duty of social insurance provision (excluding health insurance coverage) for all Iranians by merging and absorbing responsibilities and functions of SIO and the Rural Residents Social Insurance Organization. The Social Security Act also brought important changes into extent and coverage of health insurance. For example, all insured and their dependents were entitled to health services (accidents, pregnancy, and diseases, including all outpatient services, hospitalisation, pharmaceutical and diagnostic services). Also, 9% out of 30% of contribution rate was allocated for health benefits package [25, 27]. The same Act made MSSO responsible for the provision of health care services for all Iranians, including the workers (SSO was assigned to pay the whole health insurance premiums of the workers to the MSSO instead). Also, the SSO’s health care facilities were transferred to the MSSO.

In 1976, another merger occurred (the Ministry of Social Welfare and the Ministry of Wellbeing) that led to the establishment of a Ministry of Wellbeing and Welfare (Vezarat-e Behdari va Behzisti) that became responsible for the provision of health care services, social security (both insurance and supportive services), and health care insurance (except for workers). MSSO and health care facilities were merged into the Ministry of Wellbeing and Welfare accordingly. Even though the SSO was initially shut down in 1976, it was re-opened in 1979 and remained responsible for the provision of social and health insurance coverage for the workers [30]. MSSO was disbanded in December 1979 and Ministry of Wellbeing and Welfare took over the responsibility of providing health care services for the workers.

### Period 3 - Organisational solidarity in health services stewardship (1984 to 1993)

In 1985, newly established Ministry of Health and Medical Education (MoHME) replaced Ministry of Wellbeing and Welfare to solve several challenges: severe medical workforce shortage, lack of integration between the community and medical schools, slow health system dynamics and unmet community health needs. MoHME also took over the responsibility of providing health insurance coverage for the government employees and health care services for all Iranians, including workers. SSO was assigned to pay workers’ health insurance premiums directly to MoHME. As a result, for the first time, previously fragmented provision of health care services, social security, and health care insurance were unified under one ministry for all beneficiaries [25, 26]. By this time, only the government employees and workers had a stable health insurance coverage. However, there was still no reliable and defined health insurance coverage for rural dwellers and self-employed.

Over the years 1976–1989, the Ministry of Wellbeing and Welfare and MoHME were providing health care services for the SSO workers. However, officials from the SSO and the House of Workers were concerned about non-transparency of financial flows and premium utilisation. As criticisms continued, SSO officials and workers’ representatives presented to the parliament an article entitled “*The Obligation Law of the Social Security Organization*” regarding the provision of health care services for workers. Following the passing of the Obligation Law in November 1989, the SSO became directly responsible for the provision of health care services to workers and others who were subject to the Labour and Social Security laws in health facilities belonging to SSO [25–27]. Following the law, SSO could now manage the health insurance premiums of the workers by itself but also became responsible for the provision of the health care services in its health facilities or outsourcing them by contracting other public and private health facilities [27].

### Period 4 - passing universal health insurance coverage bill (1994 to 2003)

In 1990, after the end of the Iran-Iraq war, the country’s economic reconstruction led to increased health care services prices. To deal with increased prices, in 1992, the bill on Universal Health Insurance (UHI) was prepared [29, 31] and the UHI Law was passed in 1994, leading to the establishment of the Medical Services Insurance Organization (MSIO(in 1995. The main objective of UHI Act was to extend health insurance coverage for all eligible individuals within five years of enactment at most, emphasising need to provide coverage for uninsured groups, including villagers and self-employed individuals in urban areas [32]. It was also the first time the UHC through health insurance was on the policy agenda. The article 10 of UHI Law formally stipulated the need for defining the basic health insurance benefit package (BHIBP) to be provided by the basic health insurance organisations (including medical, pharmaceutical, emergency, general, specialised inpatient and outpatient medical services), as opposed to the supplementary benefits package for the commercial health insurance organisations. Before the UHI Law, there was a lack of clarity regarding which health care services should be covered by health insurance organisations [33].

To ensure the sufficient coverage of the target population and financial transparency, four separate sub-funds were created within the MSIO including Government Employees Fund, Rural Residents Fund, the Self-employed Fund and their dependents, and other sectors Funds for students, injured people in the Iran-Iraq war and some professional associations. Following the enactment of the UHI, the Imam Khomeini Relief Foundation (IKRF), which was providing support for the poor, took responsibility for the provision of health insurance coverage for its target population [26, 32].

As a result of the UHI Act, the High Council of Health Insurance (HCHI) was established, and all basic health insurance schemes including MSIO, SSO, IKRF, and Armed Forces Health Insurance Organization came to operate under the same regulations and operational instructions of HCHI. The decision on which services should be included in the BHIBP would be taken by representatives of basic health insurance organisations, medical associations, and the Medical Council of the Islamic Republic of Iran. This aimed to unify the content of the BHIBPs across basic health insurance schemes, despite having independent and separate organisational structures and financial resources [34].

Among the sub-funds of the MSIO, the financial resources of government employees were the most stable because of sustainable financial flows coming from mandatory payroll deductions. The funding for self-employed and rural residents was less stable and unpredictable. This was primarily due to the voluntary nature of health insurance coverage and lack of structure and forcible mechanisms to ensure sustainable contributions [30, 34]. As a result, rules and regulations regarding the enrolment of these two groups, premium amount, method of premiums collection, and a share of premiums to be paid by the insured (due to rising per capita health care expenditures) were changed repeatedly [34].

Until 1997, as health insurance coverage was not mandatory for the rural residents, less than 1% of 23 million rural residents were covered by the Rural Residents’ Fund. In 1997, the insurance plan for the rural and nomadic population was instigated, and 23 million people received free health insurance cards [35, 36]. It should be mentioned that per capita expenditures for rural residents were much lower than (about half of) urban counterparts. Although the coverage for the rural people was free of charge, they still had to pay in excess 25% of their health care expenditures in the case of hospitalisation. Apart from the structural and operational obstacles to collecting premiums and the extension of health insurance coverage to rural areas, the infrastructural shortages in delivering health care services in rural areas caused more difficulties for villagers attempting to access those services [36, 37]. Armed Forces Insurance Organization introduced an extra mandatory fixed health insurance premium for its beneficiaries in 2000 (so-called supplementary coverage) to include more health services in BHIBPs and provide more financial protection for its own beneficiaries and fill the gap between its expenditures and revenues. “*However, despite attempts to align the content of all BHIBPs, some differences remained. The health insurance contribution rates and health expenditure capitations were different for different groups of the population... These measures led to inequity in access to health care services over time.”* [A senior health policymaker]*.*

To solve a part of problems resulting from fragmentation in health insurance funds, the Organizing Healthcare Act (OHA) was passed in December 2002 [38]. The OHA again emphasised the importance of universal health insurance, providing equal basic benefits package for all Iranians and created a single data bank to centralise information of all population groups [39]. However, this law never came into effect due to political resistance and conflicting interests.

### Period 5 - extending health insurance coverage to all rural residents (2004 to 2013)

In 2004, a new ministry called Ministry of Welfare and Social Security (MWSS) was established to administer the three layers of the social security system including the insurance section (health, unemployment, retirement, accidents including in the workplace, basic and supplementary insurance), financial support and empowerment services for the very poor, and relief services and rescue operations in disasters. By the creation of MWSS, health insurance funds, as well as the HCHI that were operating within MoHME were transferred to the MWSS. A purchaser-provider split occurred to boost strategic purchasing of health care services and encourage competition among providers [40, 41].

In clause 91 of the fourth five-year development plan, it was determined that in order to increase the efficiency of the country’s health care system and expand and strengthen the health insurance system up to the end of 2010, the HCHI was assigned to prepare the prerequisites for the implementation of health insurance within the framework of family physician (FP) program and the referral system. The villagers’ health budget was increased fivefold with the cooperation of the Parliamentary Health Committee and Management and Planning Organization in 2005 for the development of health insurance for the rural population. This budget was given to the MSIO. MoHME seized this opportunity to implement a referral system relying on FP program for the rural residents, which had not previously been possible due to budget shortage [37, 42]. It was helpful in the reduction of inequity in health care utilisation between rural and urban areas; however, rural residents were obliged to use a referral system to access secondary health care services. About 6000 physicians and 400 midwives were added to the PHC network in three years as a part of the reform. Overall, this reform improved the access of rural residents to the hospital services and led to a modest and statistically significant increase in the hospitalisation rate and the utilisation of hospital beds [43].

When MWSS was established, it also took over from the MoHME the responsibility of defining the content of the BHIBPs. In 2008, MWSS released a new version of BHIBP formally known as a “Blue Book”. It was the first time that the contents of all BHIBPs were compiled systematically. In this book, all BHIBPs health services were categorised into nine categories: dentistry, medicine, inpatient services, outpatient services, medical equipment, medical supplies, laboratory, radiology, and physiotherapy. However, the definition of the BHIBPs content did not seem to be based on the evidence-based process or real prioritisation. Instead, BHIBPs content expansion was determined mainly by politics and the negotiations among the policy actors [33]. For example, during 2009 and 2012, multiple drug categories including 108 expensive medicines, 17 new shapes of insulin and herbal drugs became available through BHIBPs, and some copayments of prescription drugs for some specific patients were reduced. “*MSIO to a great extent depends on the public budget. It makes the financial resources more unpredictable, which in turn makes it more difficult for us to revise the BHIBPs.”* [MSIO manager]. In 2011, MWSS once again converted into the Ministry of Cooperation, Labour and Social Welfare (MoCLSW) as a part of government downsizing project.

Overall, evidence suggests that there was financial instability of the basic health insurance schemes in all three main financing functions (i.e., collecting, pooling and purchasing) [39, 44]. For example, the self-employed continued to lack reliable and sustainable coverage; health insurance coverage remained voluntary for those with no stable and regular income; there was no redistribution of cross-subsidies among health insurance funds; inequality in benefit package among different groups of the population was high; no real strategic purchasing existed. Meanwhile, OOP health care expenditure was increasing [45, 46]. As the general performance of health insurance system in the country was not satisfactory, over the time, along with the large basic health insurance funds, 17 smaller well-off institutional funds (such as banks, the Tehran Municipality, the National Broadcasting Organization, private insurance companies, the Petroleum Industry Health Organization) started to provide generous health insurance coverage for their employees independently. They did not operate under the control of HCHI and did not contribute to the general risk pooling, which had led to exacerbating the inequity in access to the health care services [47].

Difficulties in reaching UHC because of unreliable and unclear information regarding population coverage and per capita health insurance expenditure, resulting from remaining fragmented health insurance system and overlapping population statistics, and a failure to implement policy integration among insurance schemes, despite several laws being passed in this regard, led policymakers and lawmakers to put the merging of the health insurance funds on the agenda of the fifth national development plan (2010–2015) [48, 49]. It was supposed to merge all existing health insurance funds into MSIO to create a new single national scheme - the Iranian Health Insurance Organization (IHIO) that would centralise all affairs regarding health insurance. In the fifth national development plan, the government was obliged to devise required arrangements to make health insurance coverage mandatory.

As the first step in merging the SHI schemes, the charter of the IHIO was approved by the Cabinet in 2012, and the MSIO was renamed into IHIO in 2012. However, the IHIO’s establishment came to a halt at this point. The creation of a real single national insurance scheme failed due to political resistance from some of the main actors, such as the SSO and other insurance organisations, a lack of some prerequisite infrastructure, and operational challenges caused by key differences in some insurance aspects, such as financing methods, benefits packages, health service delivery, and organisational structures [17]. “*In the fifth national development plan, the method of health insurance contribution rates for IHIO beneficiaries changed from a fixed amount to 6% of salary up to 2-fold of the minimum wage.”* [A senior health insurance policymaker].

### Period 6 - extending health insurance coverage to all residents (2014 to 2020)

Despite passing several major polices over previous decades, progress towards achieving UHC was not satisfactory in all three dimensions (i.e., population coverage, health services coverage and financial support for health expenditure). Despite the repeated emphasis in the upstream documents (e.g., third to sixth five-year development plans, Mega Health Policies (2014)) on the need to extend health insurance coverage to all Iranians, reduce OOP health expenditure, and provide equitable access to health care services, self-employed continued to lack health insurance coverage and had to pay OOP for all health expenses.

According to the Statistical Centre of Iran, about 20% of Iran’s economy is informal [50]. In most cases, the informal economy sector cannot afford to pay the voluntary premium; hence, they cannot access health services. It has been one of the chronic challenges towards reaching and keeping UHC in Iran. Among those with health insurance coverage but under different health insurance schemes, there was a great inequity in terms of access to health care services due to variation in coverage, as well as amounts of coinsurances for the same services [17]. Many expensive health care services (e.g., drugs for cancer patients) were not included in the BHIBPs or access to them was impeded by high copayment. These OOP payments were especially high for beneficiaries under the coverage of IHIO and SSO. As a result, following the National Health Accounts, OOP expenses for the whole population increased from 46.2% in 2003 to 53.8% of total health care expenditures in 2008. Informal payments were common in the health system. According to the National Health Accounts, informal payments formed 14% out of 53% OOP expenditures [50]. The percentage of households facing catastrophic health expenditure varied from 8.3 to 22% [45, 51].

To overhaul the health system and also address the health financing challenges (e.g., high OOP and catastrophic health expenditure), a series of policy interventions called Health Transformation Plan (HTP) was implemented in May 2014 mainly by increasing the share of public budget for the health sector [45, 46]. The Plan’s purpose was to reduce OOP payments and financial burden in the public health sector (HTP generally does not provide financial support for patients getting health services from private health sector), by lowering coinsurance rates in the public sector and the provision of free-of-charge health insurance coverage (government paid the whole premium on behalf of the uninsured population). To achieve the aim, a new sub-fund was created within the IHIO titled “Universal Health Coverage Fund” for those who covered by the government freely. Before HTP, self-employed had to pay the half of the premium, and the government paid the rest. By launching free Universal Health Coverage Fund in 2014, self-employed individuals that were previously under the coverage of Iranian sub-fund with less than three million population shifted to this free Fund. Also, a part of SSO beneficiaries moved to IHIO to enjoy free health insurance coverage (in SSO, health insurance coverage is not delivered alone, beneficiaries should pay for other benefits such as retirement or unemployment as well).

Free Universal Health Coverage Fund expanded the basic health insurance coverage to include the 11 million previously uninsured Iranian people (mainly self-employed) which increased the overall population coverage up to 96% [52, 53]. By implementing this reform, the government announced reaching UHC by 2025. *“In HTP, health authorities decided to cover those people without coverage freely. To our surprise, in the 2016 census, 10.3 % of the population stated that they have no health insurance. That is why in the sixth national development plan, it was stipulated again that health insurance coverage should be mandatory.”* [A senior health insurance manager].

Apart from the free Universal Health Coverage Fund, another program was implemented by HTP so-called “Financial support for patients in hospitals”. It aimed to reduce the OOP expenditures in public hospitals (there is no financial support for patients getting their services in the private sector). This program which is managed directly by the MoHME provides more financial protection for patients by covering balance billing which patients paid OOP before the HTP or covering health services which are not under the coverage of existing health insurance schemes. To fund the HTP plan, the government announced that 10% of the total net of implementation of targeted subsidies law and also 1% of the value-added tax, is allocated to the health system [46]. This program aimed to increase financial access for health care utilisation so that patients do not pay more than 10% of inpatient health care expenditures. For the rural patients, this limit following the referral system was set to 5% [54]. This program ensured that patients from different health insurance schemes pay the same proportion of health expenditures which increased equity in health financing as this program provides more financial support for people with poor health insurance coverage.

Further improvements were made by revising medical tariffs and content of the BHIBP as the third phase of the HTP to improve financial protection and health equity for patients [51]. For instance, approximately 1700 previously excluded health services were added to the BHIBP and assigned medical tariffs. Additionally, to make medical tariffs more realistic in order to tackle informal payments, MoHME and HCHI revised medical tariffs fundamentally after 30 years which led to an increase in medical tariffs on average by 120% [12, 54]. Health insurance schemes coverage was also extended to include some expensive pharmaceuticals (including 53 medicines for cancer treatment). Health insurance funds became liable for the provision of additional incentives to encourage medical personnel to work in deprived and remote areas, as well as working only in public health hospitals. In June 2018, 80 cheap and common medicines were excluded from BHIBP (they are still under coverage for patients under 12).

Overall, the HTP implementation led to a sharp increase in health care utilisation due to improved financial accessibility. In deprived areas of Iran, many more hospitals and health facilities were built, and others were renovated. In the remote areas, the number of hospital beds and health care staff (physicians, nurses and midwives) was increased [55]. These improvements, alongside updating medical tariffs, led to a big increase in health care expenditures and higher costs for health insurance organisations. This led to a long delay in reimbursement to the health care providers by the health insurance funds. Gradually, concerns about the financial sustainability of the HTP forced MoHME to seek for a new solution. MoHME’s officials insisted that these challenges stemmed from the fragmentation of health insurance funds, the lack of control over the health insurance financial resources due to the purchaser-provider split, and the lack of cooperation and compliance of the SHI funds with the policies of MoHME. MoHME’s officials argue that by having the health insurance funds under their control, they can manage health financial issues better. For these reasons, as the last policy intervention in the health insurance system in 2017, IHIO was seceded from the MOCLSW and was transferred to the MoHME [56]. In the sixth national development plan (2017–2021), health insurance coverage became obligatory for all Iranians, and health insurance contribution for rural residents increased from 6 to 7% of the minimum wage. For civil servants and armed forces, it increased to 7% of their whole salary and ceiling of 2-fold of the minimum wage was removed. This enhanced equity in health financing.

Facing financial difficulties to keep free Universal Health Coverage Fund, the public budget of IHIO was diminished significantly in 2018, and the free health insurance coverage (totally financed by the government) was stopped in November 2018. New applicants were obliged to pay half of the contribution. The last project introduced in IHIO was in November 2019. According to this project, all groups enjoying free coverage (including self-employed and rural citizens) are obliged to pass a means test to assess their affordability to contribute in paying premiums or be entitled to free health insurance coverage. Those belonging to the three lowest income deciles are exempted from paying premiums, and the government will cover them, those from the fourth income decile should pay half the contribution rate for all family members, and the rest should pay the whole amount of premium out of their own OOP. Those who are eligible for free health insurance coverage will be only allowed to go to the public health sector to get health services that they need. *“One of the obstacles facing reaching UHC was free coverage for self-employed and rural citizens. The self-employed joined free Universal Health Coverage Fund in 2014 although a part of this population is rich enough to pay premiums. By launching means test for the rural citizens and the self-employed, we hope that it will enhance health equity and risk pooling by providing free coverage just for those who are extremely poor.”* [A provincial general director for IHIO]. Since Aug 2018, a new joint electronic program was introduced by the MoHME and health insurance schemes aimed to check the state of health insurance coverage of patients online and remove the duplication in health insurance coverage for the patients who have several health insurance cards.

## Discussion

According to the history of the health insurance system in Iran, and despite making great progress in all three aspects of UHC including population coverage, benefits package (covering more health services) and financial protection (increasing the depth of benefits package and financial support) as a result of health insurance reforms over the last decades especially by launching HTP to achieve UHC, there are still several unsolved challenges.

Although health insurance coverage became compulsory in Iran since the fifth national development plan, still no effective and applicable mechanisms have been introduced yet to put into effect the law of compulsory health insurance coverage for rural residents and the self-employed. It makes keeping UHC more challenging. Although HTP expanded benefits package and increased financial protection for patients in the public health sector, people (mainly IHIO and SSO’ beneficiaries) still have to pay the gap between private and public medical tariffs in the case of getting health services in the private health sector which is prevailing for outpatient services. “Financial support for patients in hospitals” program still provides inspiring extra financial protection for patients using inpatient services in the public hospitals although it stopped providing financial support for outpatient services since May 2019. Currently, it does not support those who have no insurance coverage, which can reduce health equity as, unfortunately, a considerable part of these people are from disadvantaged groups. This program also does not support patients receiving their health care services from the private health sector.

From 1972 until now, the Iranian health system has witnessed critical changes in medical tariffs. In 1992, by splitting medical tariffs into public and private sectors, the gap between the public- and private-sector tariffs and insurance coverage became more prominent. Since 2003 that Iranian Medical Council set medical tariffs for the private health sector, the private medical tariffs increased by ten times more than public health sectors and basic health insurance funds especially IHIO and SSO failed to cover this gap. This reduced the access to health care services and increased OOP expenditures in private health sector [51]. Health insurance funds should expand their coverage in a way to reduce household expenditures in the private health sector and remove the direct payment between patients and healthcare providers [57].

For decades, the main focus of health insurance reforms was on extending population coverage rather than increasing health services coverage or enhancing financial protection [41]. Apart from unmet health needs, a weak benefits package and low financial protections resulted in high OOP expenditure and the presence of informal payments [10, 11].

### Fragmentation in the pooling of health insurance funds

Even though the physical integration of the existing health insurance funds and the formation of a single national insurance scheme were legislated in 2010, they were never implemented in practice. Reducing fragmentation by creating a single national scheme could have facilitated moving toward reaching UHC and improving health equity by controlling total health care expenditures, implementing strategic purchasing, better supervision of health care providers by centralizing their profiles in a single database, reducing fraud and controlling the volume of provided health care services, improving the health financing equity by setting the same coinsurance rates for different population groups and boosting risk pooling for the whole population, eliminating duplication in population coverage, centralizing health profiles and health expenditure profiles of beneficiaries in a single database [58]. It is worth mentioning that according to Mega Health Policies approved in 2014, health financial resources should be managed by the health insurance system. However, currently, a part of government subsidies to provide financial protection for the patient (including the program of “Financial support for patients in hospitals” and drug subsidies to reduce coinsurance for patients with chronic and expensive diseases) are managed by the HTP and Deputy for Food and Drug respectively rather than health insurance system.

### The high financial burden of free health insurance coverage for the poor, rural residents and self-employed persons

Providing UHC for the self-employed is still a major challenge in Iran, even 26 years after the passing of the UHI law. The fact that insurance coverage is non-compulsory, the large scale of the informal economy in Iran (about 20%), the financial inability of people to pay the premiums, the shortages and instability of government financial support for the constant development of insurance coverage for villagers and the self-employed, as well as not devising reliable methods to engage rural people and self-employed in paying at least a part of premiums are among the main reasons for not achieving extending coverage for all self-employed. Apart from poor people, providing free health insurance coverage for about 23 million rural citizens and 11 million self-employed persons have imposed a high financial burden on the government and jeopardise the sustainability of UIC in Iran. By launching free Universal Health Coverage Fund in 2014, those who were participating in paying health insurance premiums including the self-employed and a part of SSO beneficiaries with voluntarily based coverage moved to IHIO to enjoy free health insurance coverage. In reality, just a part of 11 million persons covered by this project was new and did not have previous insurance coverage. This harmed moving towards reaching UHC and health equity, as those who got used to paying premiums shifted to free coverage again, and no distinguishing method was applied to extend health insurance coverage just for real underprivileged groups. Introduction of the means-testing project by IHIO in November 2019, can improve health equity by targeting those people in need although according to the current results of the means-testing project, majority of rural dwellers and self-employed still should be covered freely by the government. This program is at its beginning, and how it is going to affect the UHC is not clear. Although alongside the ability to pay, it should be mandatory for all to get health insurance coverage, which remains to be challenging.

Lessons from South Korea show that a stable national economic growth has been an inseparable contributor for extension of health insurance coverage for the self-employed, the last group that joint national insurance coverage. Rapid considerable economic growth in late 1980 made it possible for the government, employers and the self-employed to invest in and pay for the health insurance [59, 60]. In Turkey, high economic growth from 2003 to 2012 enabled the government to invest in the health sector and spend more money on social services [7].

### Need to constrain total health care expenditures

Joint work between two ministries of MoHME and MOCLSW to control total health care expenditures is vital if reaching and keeping UHC is a priority for both. By curbing health care expenditure, it is possible to lower the health insurance premiums, which enables more people to buy health insurance coverage. It will also enable health insurance schemes to include more health services in their benefits package and provide more financial support for the services they cover. Moreover, it will allow the government to pay premiums for more people (particularly, for most disadvantaged). Reaching UHC also needs working on supply-side (constraining the amount of health care services provided in the health system) alongside reforms in the demand side (health insurance system reforms) to control health care expenditures. The amount of money injected in the health system by the HTP was unprecedented and ambitious projects were implemented in both the health insurance side and also health care provision side. Governmental health expenditures per cent out of government budget increased from 11.4% in 2013 to 19% in 2016 [46]. However, as spending-cuts policies were not applied simultaneously, increasing health expenditures jeopardised the financial sustainability of HTP, and in turn, achievements attained in UHC became hard to sustain.

### Study strengths and limitation

To our knowledge, this is the first study conducted in Iran comprehensively explore the health insurance policy trends and milestones through multi methods. The main limitation of the study was lack of enough published documents and also low information of interviewees regarding first and second periods of the study to cover these periods in more details.

## Conclusion and policy recommendations

There is no single way of providing universal health insurance coverage for all population. Providing access to health services and financial protection against health costs, for everyone, can be a long-term process. Countries should focus on creating a clear role for public revenues in the health system financing while understanding the limitations of relying on the ability of the population to afford to pay insurance premiums. We strongly believe that to reach and keep UHC in Iran, the two main players of the health insurance system (MoHME and MOCLSW) should devise and follow the same policies to control health care expenditures. To do so, they can implement policies, such as extending family physician programme to all population groups, implement and apply close-ended payment methods, use clinical guidelines, revise the content of the benefits packages by including the most cost-effective interventions, devise more restrictive regulations to rationalise the prescription of diagnostic services, control the price of pharmaceuticals and medical supplies, control the high technologies/population ratio, and control the moral hazards by setting more realistic coinsurance rates for the programs under the coverage of HTP.

Without controlling total expenditure, government, and health insurance funds like IHIO have to abolish free coverage for segments of the population, omit some health care services from the benefits packages or impose more financial burden on the patients at the service utilisation point which can lead to higher OOP expenditures, reduce health equity and paralyse keeping UHC. Majority of outpatient and diagnostic health services in Iran is provided by the private health sector, which is not under the coverage of HTP and is one of the main sources of increasing OOP payments. The government should extend the capacity of providing outpatient and diagnostic health services in the public health sector and support patients financially in the deprived cities for necessary health services which are available just in the private sector. Working on the medical tariffs and closing the gap between private and public medical tariffs should be considered to make UHC in Iran more sustainable. The compulsory health insurance coverage law still needs to be implemented in full. Also, it would be necessary to assess and analyse the impacts of health insurance and health financing reforms in Iran in terms of improving financial equity indicators, financial sustainability, equity in utilisation and access to health care services for disadvantaged groups, technical and allocative efficiency. Finally, we should assess the short and long-term results of IHIO means-testing project on UHC. Paying health premiums and providing free health insurance coverage may be difficult for people (particularly for rural citizens who have enjoyed free coverage for over 26 years) and government due to the current tough economic situation in Iran caused by the international sanctions and also by mass unemployment caused by COVID-19 pandemic.

## Data Availability

Not applicable.

## Abbreviations

UHC: Universal health coverage
GDP: Gross National Income
PPP: purchasing power parity
PHC: Primary healthcare
SHI: Social health insurance
UIC: Universal insurance coverage
MSSO: Medical Services Security Organization
SSO: Social Security Organization
MOHME: Ministry of Health and Medical Education
UHI: Universal Health Insurance
IKRF: Imam Khomeini Relief Foundation
HCHI: High Council of Health Insurance
OHA: Organizing Healthcare Act
CWSSS: Comprehensive Welfare and Social Security System Law
MWSS: Ministry of Welfare and Social Security
MOCLSW: Ministry of Cooperation, Labour and Social Welfare
FP: Family Physician
IHIO: Iranian Health Insurance Organization
CHE: Catastrophic health care expenditures
HTP: Health Transformation Plan
